# Nitrites in Cured Meats, Health Risk Issues, Alternatives to Nitrites: A Review

**DOI:** 10.3390/foods11213355

**Published:** 2022-10-25

**Authors:** Mynul Hasan Shakil, Anuva Talukder Trisha, Mizanur Rahman, Suvro Talukdar, Rovina Kobun, Nurul Huda, Wahidu Zzaman

**Affiliations:** 1 Department of Food Engineering and Tea Technology, Shahjalal University of Science and Technology, Sylhet 3114, Bangladesh; 2 Faculty of Food Science and Nutrition, Universiti Malaysia Sabah, Kota Kinabalu 88400, Sabah, Malaysia

**Keywords:** cured meat, nitrites, natural alternatives, health concerns

## Abstract

Nitrite is one of the most widely used curing ingredients in meat industries. Nitrites have numerous useful applications in cured meats and a vital component in giving cured meats their unique characteristics, such as their pink color and savory flavor. Nitrites are used to suppress the oxidation of lipid and protein in meat products and to limit the growth of pathogenic microorganisms such as *Clostridium botulinum*. Synthetic nitrite is frequently utilized for curing due to its low expenses and easier applications to meat. However, it is linked to the production of nitrosamines, which has raised several health concerns among consumers regarding its usage in meat products. Consumer desire for healthier meat products prepared with natural nitrite sources has increased due to a rising awareness regarding the application of synthetic nitrites. However, it is important to understand the various activities of nitrite in meat curing for developing novel substitutes of nitrites. This review emphasizes on the effects of nitrite usage in meat and highlights the role of nitrite in the production of carcinogenic nitrosamines as well as possible nitrite substitutes from natural resources explored also.

## 1. Introduction

Meat curing is an ancient method of food preservation that is still widely used today [1]. It refers to adding nitrite/nitrate salt, common salt (NaCl), and spices to fresh meat in varying degrees of comminution and at various processing phases [2]. Prior to the invention of refrigeration, meat was preserved using methods discovered to be efficient in controlling deterioration after slaughter and extending the food supply during times of shortage. Despite being lost in time, the curing process is thought to be evolved from salt preservation methods as early as 3000 B.C. [3].

Among the various additives used in meat curing, nitrite salt is very significant. Nitrite is a major intermediary throughout the biological N-cycle present in soil and water surface [4]. It’s a versatile chemical with a wide range of uses, including dye manufacturing and food preservation [5]. Nitrites in various meat products are significant preservatives and impede the growth of several unwanted micro-organisms [6,7]. Nitrite is added to cured meat at levels less than 150 ppm to prevent the development of microbiological organisms like *Clostridium botulinum*, which causes food poisoning [8].The main reasons for using nitrite as a preservative in meat are: (1) To inhibit the *Clostridium botulinum* from spreading and secreting toxins that cause food toxicity [9,10]. (2) to provide the necessary bright red color in meat products like sausage, ham, salami, etc. [11]. (3) To give cured meats their characteristic texture and aroma [12]. In addition, nitrite also inhibits the oxidation of lipids in meat products and thus prevents rancidity (off flavor) [13].

Since the middle of the 1980s, research has shown that nitrite is a major chemical with substantial impacts on human health. Vegetables are a great source of dietary nitrates, and they have been proven to be an important source of endogenous nitrite as well as nitric oxide (NO) in the human body [14]. Nitric oxide (NO), produced through enzymatic synthesis, regulates blood pressure, wound healing, immunological response and neurological processes in the human body [15]. New research has demonstrated that NO (nitric oxide) regulates blood circulation in cardiac tissues and perhaps in other body tissues [16,17]. Furthermore, regular nitric oxide and nitrite production may help to prevent cardiovascular diseases like hypertension, atherosclerosis, and stroke [18].

High nitrite concentrations, on the other hand, are extremely dangerous for infants since they can develop an infant’s methemoglobinemia [19]. Furthermore, cancer-causing nitrosamines are formed when nitrite reacts with secondary or tertiary amines [20]. Recently, the International Agency for Research on Cancer (IARC) stated processed meat as carcinogenic by evaluating sufficient epidemiological data [21]. IARC also reported that ingested nitrite from processed meat can lead to colorectal cancer in human. Because of these harmful effects, many countries have severely restricted their use on processed food products [22]. The toxicity of nitrite is ten times that of nitrate. For humans, the fatal oral dosage is 80 to 800 mg nitrate per Kg body weight and just 33 to 250 mg nitrite per Kg body weight. Long-term intake of increasing amounts of red meat, especially processed meat, is linked to a higher rate of mortality, colorectal cancer, type-2 diabetes and heart diseases in both male and female, according to large prospective United States, E.U. cohort studies as well as meta-analyses of epidemiology [23]. Concerning these issues, an acceptable daily intake (ADI) of 0.07 mg nitrite per kg of body weight was set by the Joint Expert Committee of the Food and Agriculture Organization (JECFA) and the World Health Organization (WHO) that appears to be safe for healthy newborns, children, and adults [24].

The World Cancer Research Fund (WCRF) and the American Institute for Cancer Research (AICR) published research in 2007 that found a moderate but significant link between increasing consumption of processed meat and a higher risk of colorectal cancer [25]. As a result, specialists advised limiting red and processed meat consumption. Anywise, eating less meat alone may not result in a significant reduction in carcinogenic effects and it may be associated with several disadvantages, including the loss of nutritive value, especially iron (Fe). So, it will be wise to invest proactively in the processing of healthier meat products rather than anticipating processed meat consumption to fall.

Because of growing concerns regarding sodium nitrite’s long-term adverse effects, their use in cured meat products is strictly regulated among most developed countries. Parallel to this, the new ideas of all-natural and clean label have raised a demand for healthy and high-quality meat products. Due to health hazards, consumers choose natural additives over chemicals in processed meat. As a result, research on substituting natural ingredients for the chemical additive nitrite has grown over the years. To reduce the risk of nitrosamine formation and mitigate potential human health hazards, researchers are trying to find effective ways in meat curing. One such method is the replacement of nitrite salt with alternative ingredients that have similar properties while posing no health risk [26]. However, no single alternative that provides the multi-functions of nitrite in meat products has yet been found. The employment of “hurdle technology” in meat curing is one proposed solution to this issue where low amounts of nitrite are mixed with other ingredients [27].

The aim of this present work is to review the role of nitrite in cured meat products, the adverse health effects of higher nitrite intake as well as to give an overview of the available data on potential replacements to nitrite salt in processed meat either whole or partially.

## 2. Methods

### 2.1. Searching Strategy

The required information for the current study were collected by searching on popular databases such as Google scholar, PubMed and Scopus. About 84 published research and articles ranging from 2000 to 2021 in English were collected and analyzed for this review paper.

### 2.2. Study Selection and Inclusion, Exclusion Criteria

Articles were searched using the following keywords such as “nitrite”, “meat”, “health effect” and “alternatives”. Among all the articles found related to these keywords and time frame, articles related to detection and removal of nitrite from meat products were excluded. On the other hand, articles related to the health effect of nitrite on human health and its possible alternatives were included. Studies related to the adverse health effects of nitrite on animals, or other organisms were excluded.

### 2.3. Data Extraction

**Figure 1:**
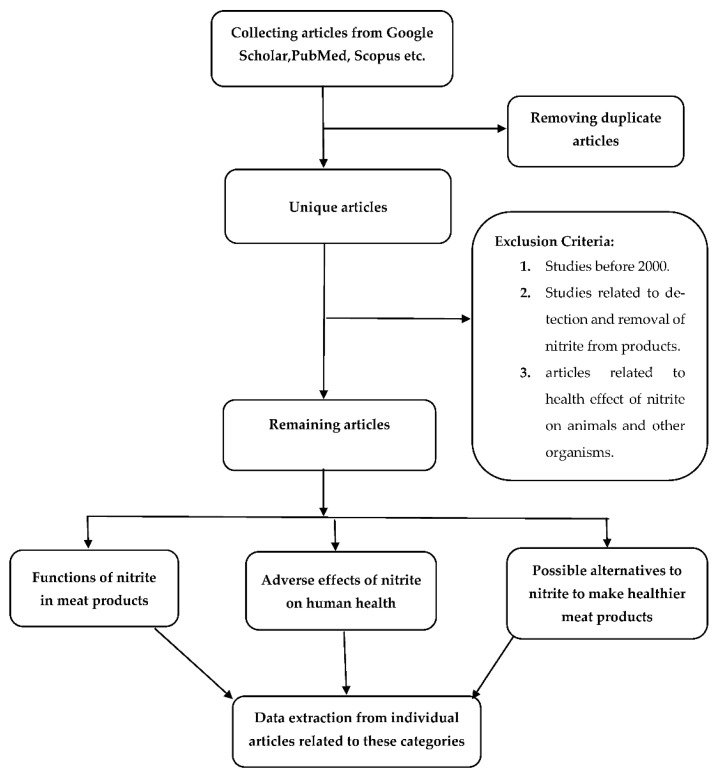
Flow diagram of the literature search and study choosing procedure.

Reliable and consistent data from individual studies related to nitrite functions on meat products, its adverse effect on human health and its alternatives were extracted for making this review paper. The following Figure 1 shows the flowchart of this study:

## 3. Sources of Nitrite

Nitrites play a vital role in the biogeochemical cycle of nitrogen in natural water. They can be found in soils [28], waterways [29], foodstuffs [30], plants [31], air (as nitrogen dioxide) [32], and biological samples [33].

**Figure 2:**
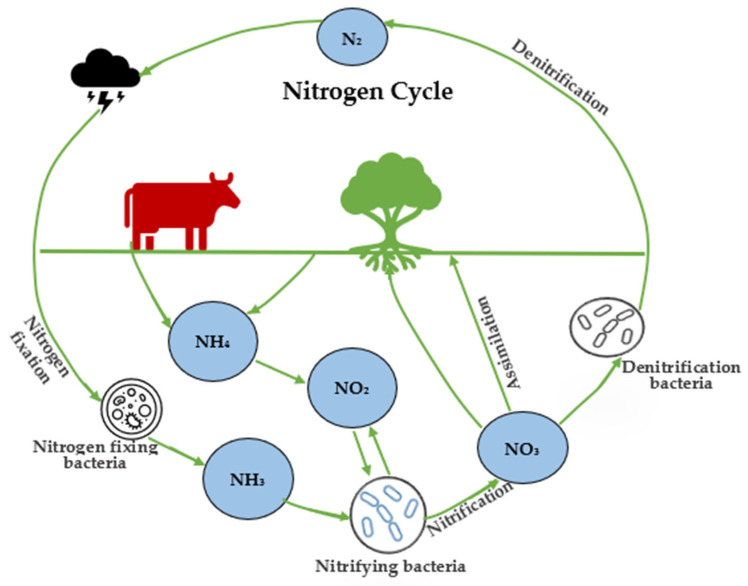
Nitrogen cycle in the environment including Nitrogen Assimilation.

In the biological nitrogen cycle, nitrogen is converted to nitrate by bacteria, which is taken by plants and incorporated into tissues (Figure 2). Animals that consume plants utilize nitrate to produce proteins. Animal excrement and microbial breakdown of animals and plants after death return nitrate to the environment. Nitrate or the ammonium ion can be converted to nitrite by micro-organisms; this reaction occurs in the environment, digestive tracts of humans and other animals. Once bacteria in the environment convert nitrate to nitrite and subsequently convert the nitrite to nitrogen, the cycle is completed.

In the entire nitrogen cycle, nitrite is the intermediary compound derived from nitrification. The microbiological process, carried out successively by multiple species of bacteria (*Nitrosomonas* and *Nitrobacter*), which converts ammonium to nitrates via the intermediate production of nitrite, is called nitrification [34]. The following are the two steps in the nitrification process, along with their equations:

(1) *Nitrosomonas* bacteria converts ammonia to nitrite
  2NH_4_^+^+ 3O_2_ → 2NO_2_^−^ + 4H^+^ + 2H_2_O (1)
(2) The nitrite is converted to nitrate by *Nitrobacter* bacteria [35].
  2NO_2_^−^ + O_2_ → 2NO_3_^−^ (2)

## 4. Function of Nitrite in Cured Meats

### 4.1. Cured Color Development

Meat color is highly variable and is influenced by a variety of factors. When nitrite is introduced to meat, it is converted to nitric oxide (NO) via the reactions listed below [36,37]:

(1) NO_2_^−^ + H^+^ ↔ HNO_2_
(2) 2HNO_2_ ↔ N_2_O_3_ + H_2_O
(3) N_2_O_3_ ↔ NO + NO_2_

Here, nitrite reacts with hydrogen ions (H^+^) of water to produce nitrous acid. After that, nitrous acid progressively decomposes into water molecules (H_2_O) and dinitrogen-trioxide (Equations (1) and (2)). Then, nitric oxide and nitrogen dioxide are generated from dinitrogen trioxide (N_2_O_3_). The major component responsible for nitrite’s apparent function in cured meat products is nitric oxide.

**Figure 3:**
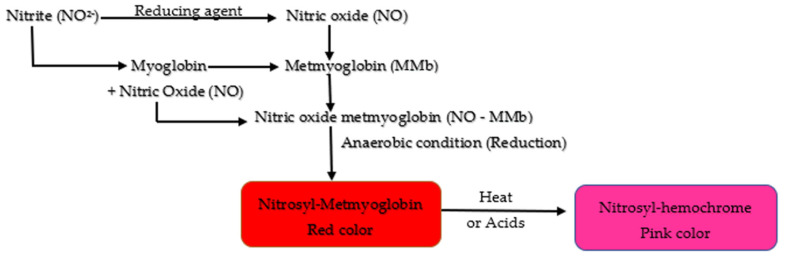
Mechanism of color development in cured meats.

Nitric oxide combines with the iron of both myoglobin (Fe^2+^) and metmyoglobin (Fe^3+^) to produce a cured pink color in meat [27]. Myoglobin is the sarcoplasmic protein responsible for the red color in meat, and metmyoglobin (brown in color) is the oxidized form of myoglobin (Mb). NO-myoglobin is formed when nitric oxide (NO) reacts with myoglobin (Fe^2+^). The bright red nitrosyl-myoglobin complex provides the foundation for the distinct color of cured meat [38]. This complex is extremely unstable, and it turns into a stable, eye-catching reddish-pink pigment (nitroso-hemochrome) during heat treatment (Figure 3).

Furthermore, myoglobin may react with HNO_2_. Myoglobin (Fe^2+^) combines with nitrous acid and forms metmyoglobin (Fe^3+^) by oxidation. Metmyoglobin (Fe^3+^) then reacts with NO to produce NO-metmyoglobin. NO-metmyoglobin is also produced from the reduction of metmyoglobin [39]. As a result, the meat becomes brown in color. NO-metmyoglobin can be converted to NO-myoglobin by a reductant, causing the formation of the cured color (pink) again when heated.

The presence of other additives in cured meats also affects the color development. Antioxidants including erythorbate, ascorbic acid and polyphenols stimulate the production of NO by allowing the N_2_O_3_ reduction [40]. Ascorbic acid reduces Fe^3+^ to Fe^2+^ effectively and enhances the reduction process of NO-metmyoglobin [27]. Thus, antioxidants with reducing activity aids in the cured meat color development by raising NO production and lowering NO-metmyoglobin levels. NaCl, generally added to meat for curing, reacts with HNO_2_ to generate nitrosyl chloride, which is more sensitive than N_2_O_3_ in terms of generating nitric oxide (NO) and initiating the formation of NO-myoglobin [41].

The rate of nitrosyl myoglobin production has been found to increase with increased salt concentration [42]. The sensory panelists rated the bacon with a high sodium chloride level as having more redness [43]. The pH also controls nitric oxide formation from nitrite. Nitrous acid (HNO_2_) and nitrite reactivity increase as pH decreases [44]. The rate of nitric oxide (NO) formation is doubled when the pH is slightly reduced by 0.2–0.3 units [45].

Basically, a very small quantity of nitrite is required for the development of the cured color in meats, usually approximately 2–14 ppm [26]. However, the level of residual nitrite in cured meats gradually decrease owing to oxidation during storage time. As a result, the meat starts to lose its cured color and become faded. Color loss also occurs when meat is exposed to air and light, while the presence of adequate residual nitrite as well as reducing chemicals delay this process [46]. Usually, 10–15 ppm of residual nitrite is recommended, which can act like a reservoir for the cured meat color regeneration [26]. On the other side, higher levels of sodium nitrite (>600 ppm/kg of meat) and low pH value may lead to nitrite burn (discoloration) where meat shows a green color due to the formation of nitrihemin, a green-brown pigment [47].

### 4.2. Cured Flavour Development

Flavor is the combination of numerous qualities including odor, fragrance, taste, texture and temperature of meat that influences the perception of the consumer [48,49]. Although it is generally recognized that nitrite influences the meat flavor, the reactions responsible for this thing are not completely understood.

The antioxidant activity of nitrite against lipid oxidation is assumed to be one of the methods which might alter the flavor of meat products by suppressing “warmed-over” flavor. Aldehydes such as pentanal, hexanal, etc., which are the products of lipid oxidation, are suppressed in cured meat when lipid oxidation is inhibited by nitrite [49]. Uncured meat has considerably greater levels of hexanal than cured meat. Furthermore, cured meat has low levels of carbonyl compounds, including 2-heptanone, 3-hexanone, 2-nonenal, and 2-octanal [50]. Thus, nitrite has been demonstrated to simplify the flavor spectrum. The use of nitrite does not affect the synthesis of specific flavor compounds, but it inhibits the formation of aldehydes (hexanal), masking the sulfur-containing chemicals that give cured meat its flavor.

Nitrite, on the other hand, has been shown to cause the production of Strecker aldehydes. Strecker aldehydes are generated when amino acids are degraded by dicarbonyl produced through Maillard reactions and these aldehydes are linked to meat flavor formation [51,52]. After adding nitrite to fermented sausages, the production of Strecker aldehydes increases. This might be due to an increase in carbonyl molecules, which can combine with amino acids to create Strecker aldehydes due to the pro-oxidant action of nitrite [52].

In cured meats, less than ^1^/_2_ of the overall volatile chemicals, generally found in uncured meats have been detected and much of the variation is considered to be related to the partial production of the by-products of lipid oxidation. Alcohols and phenolic compounds may go through nitration reactions, which may have an effect on volatile chemicals. S-nitroso thiol production and disulfide bond breakdown during meat curing is likely to cause increases in sulfur compounds. The antioxidant effect of nitrite explains why oxidation products, such as hexanal, are reduced in cured meats. More research is needed to completely understand the mechanism, reactions and the volatile compounds responsible for the aroma and flavor of cured meat [46].

Sensorial research shows that cured meat flavor is not only an outcome of the retardation of lipid oxidation but also a blending of complex cured aromas/flavors in collaboration with the scarcity of rancid flavors [53]. In this manner, it can be said that cured meat flavor is the combination of two things:

✓ Lipid oxidation suppression by nitrite;
✓ Nitrite related flavor development.

### 4.3. Antioxidant Properties against Lipid and Protein Oxidation

Another notable characteristic of nitrite is that it can prevent rancidity during storage and the formation of “warmed-over” flavors when meat products are heated [38]. The oxidation process affects lipids, proteins as well as pigments of meat and causes changes in hue, flavor, texture, and nutritive value [54]. During cold storage, lipid oxidation produces off-flavors which are typically characterized as rancid and enhances the discoloration of food [55]. Moreover, it produces and accumulates chemicals that might endanger consumers’ health [54]. Oxygen is a significant factor influencing lipid oxidation in meat. It interacts with the unsaturated lipids of meat to generate lipid peroxides which include oxygen absorption as well as double bond reformation [56]. The production of lipid peroxides ultimately leads to the formation of a variety of chemical components such as aldehydes, alcohols and ketones [54].

Nitrite acts as an antioxidant by protecting the lipid molecules of meat from oxidation. In cured meats, nitrite works as an antioxidant through different mechanisms. Nitrite serves as a chelating agent of metallic ions (main prooxidants in meats) and it also stabilizes the heme Fe [57,58]. Furthermore, nitric oxide, produced from nitrite, may be readily converted to NO_2_ by reacting with oxygen [36]. Nitric oxide also reacts with radicals of lipid to break the oxidation chain reactions [44]. Lipid oxidation may be started in a variety of ways and once initiated, grows exponentially due to free radical interactions. Once they are generated in the starting phase, lipid radicals are continually oxidized through radical chain reactions. Nitrite can inhibit lipid oxidation initiation by reacting with ROS (reactive oxygen species), such as hydroxyl radicals. Nitric oxide (NO) can also inhibit lipid oxidation by combining with lipid peroxyl radicals and produce non-radical molecules. Nitrite has been shown to have an antioxidant property at concentrations as low as 40 mg per kg [59]. A reduction of about 65% in lipid oxidation has been reported when 50 ppm sodium nitrite was added to the meat products [60].

Proteins, in addition to lipids, are oxidized during the preparation of meat. Moreover, the antioxidant action of nitrite in the inhibition of protein oxidation is yet unknown. As the protein oxidation mechanism is similar to the mechanism of lipid oxidation, it is believed that nitrite might hinder protein oxidation. The quantity of peroxide value, sulfhydryl, carbonyl groups and thiobarbituric acid-reactive compounds (TBARS) produced during meat processing are commonly used to assess meat oxidation [61]. The application of sodium nitrite to meat products results in a considerably lower TBARS value than that of controls (without sodium nitrite), but no influence on the carbonyl compound concentration, used to evaluate protein oxidation [62]. Sodium nitrite has been shown to have both antioxidant and pro-oxidant properties in meat products. As evidenced by the decreased generation of carbonyl compounds, sodium nitrite exhibits an antioxidant property towards protein oxidation. However, nitrite was also discovered to possess a pro-oxidant effect on protein oxidation by lowering the total sulfhydryl concentration and increasing disulfide bond formation in cooked sausage proteins. By absorbing oxygen from sensitive molecules or producing reactive nitrogen species, nitrite can serve as a pro-oxidant [63]. Protein oxidation causes a variety of physicochemical as well as nutritional changes in meat proteins along with a reduction in amino acid bioavailability, difference in composition of amino acids, decline in protein solubility, reduction in protein digestibility and lack of proteolytic activity [64]. All these changes can be minimized by the antioxidant activity of nitrite. Therefore, it can be said that nitrite plays a great role as an antioxidant by inhibiting lipid and protein oxidation and thus it can prevent meat quality deterioration.

### 4.4. Antimicrobial Effect

Nitrite has been found to be very effective as a bacteriostatic and bactericidal agent in inhibiting or regulating the development of bacteria to various degrees in meat products. Nitrite has been shown to impede the reproduction of *Clostridium botulinum*. The application of nitrite has been shown to inhibit the formation of botulinal toxins from inoculated *Clostridium botulinum* in wiener sausages during storage. There are two effects of nitrite found in controlling the growth of *Clostridium botulinum*. The first effect is inhibiting vegetative cells developing from surviving spores. The second effect is the prevention of vegetative cell division [9,65]. During meat preservation, nitrite lowers the amount of *Clostridium sporogenes*, which have comparable characteristics to *Clostridium botulinum*. In addition, numerous studies have found that nitrite inhibits the development of *Listeria monocytogenes*, *Bacillus cereus*, *Clostridium perfringens* and *Staphylococcus aureus* in various meat products [38,66]. The impact of nitrite and inhibitory mechanisms varies with several bacterial species [67]. The effectiveness of antimicrobial activity is dependent on various factors like pH, residual nitrite level, salt concentration, Fe content, reductants presence, storage temperature, etc. [68]. At acidic pH, nitrite hinders the growth of unwanted microorganisms more effectively [69]. 

Nitrite attacks bacteria at numerous sites by blocking metabolic enzymes, restricting oxygen absorption, and breaking the gradient of protons. Furthermore, nitric oxide binds to iron and reduces the availability of iron which is required for enzyme activity as well as bacterial metabolic activity and development [70]. Because of the strong reactivity of Fe and nitrite, heme ion centers of enzymes and Fe-sulfur complexes are the major target of nitrite. The antibacterial activity of nitrite may be due to the peroxynitrite (ONOO) formation and nitric oxide formation from nitrite [71]. Acid catalysis may cause oxymyoglobin to be autoxidized, generating superoxide radicals. The interaction of nitric oxide with superoxide radicals as well as the reaction of nitrite with hydrogen peroxide can produce peroxynitrite. Under physiological environments, peroxynitrite and peroxynitrous acid (ONOOH) stay in equilibrium. These two compounds are strong oxidants as well as nitrating agents [72]. They penetrate the bacterial cells by passive anionic diffusion and disrupt the microorganisms by causing protein and lipid oxidation or by damaging DNA [72,73]. Nitric oxide (NO) can also inhibit microbial growth by forming protein-bound dinitrosyl iron complexes when it reacts with iron-sulfur proteins, which are engaged in critical physiological activities including energy metabolism & DNA synthesis [74].

Various kinds of microorganisms have various metabolic pathways and antioxidant defense strategies, and certain microorganisms are found to be resistant to the oxidative stress of peroxynitrite and peroxynitrous acid [71]. Furthermore, the antibacterial action of nitrite in Gram-positive anaerobic bacteria has been shown to be more effective than in Gram-negative aerobic bacteria.

Most of the nitrite applied to cured meat products is used to suppress *C. botulinum*, with only a little amount (about 25 ppm) required for color development. Suppression of *C. botulinum* development and toxin generation rises when nitrite levels rise. The level of additional nitrite is thought to have a greater influence on inhibiting *C. botulinum* than that of the residual nitrite during storage, implying that the production of antimicrobial compounds as a consequence of nitrite-related reactions might be noteworthy [75]. The growth of starter cultures and bacteriocin production have been shown to be inhibited when the nitrite concentration was 100 ppm in sausage (fermented using *Lactococcus lactis*). An estimation predicts that when the nitrite content in sausage fermented with *Lactococcus lactis* reached 100 ppm, the development of starter cultures and bacteriocin synthesis were suppressed [76]. Several other estimates suggest that pathogens including *Listeria monocytogenes*, *Staphylococcus aureus*, *Bacillus cereus* and *E. coli* grow slower in the presence of nitrite at levels found in cured meats and poultry products [17].

## 5. Health Concerns Associated with Nitrite in Meat

Despite all of sodium nitrite’s benefits, its use in meat has been a bone of contention. Due to nitrite’s high chemical reactivity, it can combine with a variety of components in meat systems. The heat used throughout the thermal treatment of cured meat products increases its reactivity. Particularly, nitrite ions are highly reactive when the pH is lower than 7; it may react with a variety of meat components, including amino acids, sulfhydryl, amines, phenolic compounds, ascorbic acid and myoglobin. Nitrite can play a role as a nitrosating agent and form various nitroso compounds [77]. Other nitrosating agents include nitrous acid and nitric oxide which are also derived from nitrite. Nitrous acid participates in the processes that result in the formation of endogenous N-nitroso compounds (NOCs). NO, on the other hand, maybe a generator of nitrates and nitrites, which circulate in the body of human [78]. Generally, N-nitroso compounds are classified into six types: non-volatile N-nitrosamines, volatile N-nitrosamines, N-nitrosated heterocyclic carboxylic products, N-nitrosamides, Amadori compounds and N-nitrosated glycosylamines [79]. The majority of volatile nitrosamines are categorized in group 2B, which means they are potentially carcinogenic to the human body [80]. The number of nitrosamines in processed meat products varies depending on the type of meat product. The quantity of N-nitrosamines in processed meat might be less than the detection limit (one microgram per kilogram) [81]. Furthermore, NOCs are formed when food is cooked at high temperatures or when cured meat is processed. Recent epidemiologic studies have indicated nitrate, nitrite and N-nitroso compounds as a potential risk for cancer [82]. Among the various nitroso compounds, N-nitroso dimethylamine is thought to be potentially more carcinogenic to the human body. Although nitrite is known to be associated with general health implications, no evidence has been found to support the connection between cancer risk and processed meats consumption [82]. Only high exposure to nitrites from various sources has been attributed to the elevated risk of health problems [83].

As sodium nitrite can be a predecessor of nitrosamines, its usage in meat curing has gathered public concern. It is currently considered that the amount of nitrite added and the production of N-nitrosamines have a positive relationship but the relationship is not linear [84]. The majority of N-nitrosamines are organ-specific, implying that only certain types of them cause cancer in certain organs [18]. Furthermore, they exhibit teratogenic effects too. There are about 300 variety of nitrosamines and almost all of them (97%) have been demonstrated to be teratogenic in experimental animals [85]. Amines, in the form of free amino acids (proline, hydroxyproline), creatinine and creatine are present at very low concentrations in organic meat products [36]. 

The development of nitrosamines in meat products is a complicated process and it may be influenced by a wide range of factors. Nitrite, nitrate, primary and secondary amines, amides, peptides, proteins and various amino acids are the initial compounds for N.A. synthesis in meats and these are converted into N.A. (nitrosamines) precursors by microbial activity. Microorganisms may contribute to the formation of N.A.s by converting nitrates to nitrites and degrading proteins to amino acids and amines [86]. N-nitrosamines can develop in meat throughout the production processes, during home cooking and in the digestive tract after ingestion [87]. They are mostly generated from secondary amines, nitrite and other nitrosating agents. In cured meats, residual nitrite may combine with amines and free amino acids and yield nitrosamines under specific conditions, such as the existence of secondary amines, low pH, product temperature >130 °C and the NO_2_ availability to react [78]. During the grilling or frying of cured meats, nitrosamines may occur in little amounts and are expected to cause cancer in the human body (even with the little exposure over prolonged time) [88]. 

The chemical reactions that result in the developments of nitrosamines in cured meat systems are noted below:

NaNO_2_ + H^+^ → Na^+^ + HNO_2_

HNO_2_ + H^+^ → NO^+^ + H_2_O

2HNO_2_ → N_2_O_3_ + H_2_O

N_2_O_3_ → NO + NO_2_

NO + M^+^ → NO^+^ + M

RNH_2_ (Primary amine) + NO^+^ → RNH-N = O + H^+^ → ROH + N_2_

R_2_NH (Secondary amine) + NO^+^ → R_2_N-N = O + H^+^

R_3_N (Tertiary amine) + NO^+^ → no nitrosamine formation

These chemical reactions exhibit the same process leading to the formation of nitric oxide and nitrous acid. As a result, the same consequences can lead to the nitrite reduction and favors the production of nitrosamine. Among the primary, secondary and tertiary amines, the secondary amines generate more persistent nitrosamines. In addition, the mixture of secondary amines and nitrite cause lung adenomas in mice. An investigation into mice treated with 0.5% sodium nitrite and 0.85% butyl urea showed the elevated occurrence of malignant lymphomas. Numerous epidemiological studies have found a link between nitrosamines (N.A.s) and various type of cancer risk [89,90,91,92]. In 2006, a working group of IARC (International Agency for Research on Cancer) stated that “ingested nitrite under certain conditions resulting in endogenous nitrosation is presumably carcinogenic to human body” [93,94]. An epidemiological study conducted in 2008 showed that there is an increased risk of colorectal cancer related to high processed meat intake [95]. Excessive nitrite intake can also result in tissue poisoning, respiratory center paralysis, and other hypoxia-related symptoms. In extreme cases, it can cause suffocation as well as death by decreasing the O_2_ carrying capability of hemoglobin in human blood [96]. High nitrite consumption can impair iodine metabolism and decrease iodine absorption by the thyroid, which can result in the enlargement of the thyroid gland.

Methemoglobinemia, also known as “blue baby syndrome”, is another health concern of high nitrite intake. It develops when nitrate is converted to reactive nitrite by reducing bacteria in the saliva or digestive system of humans. The blue baby syndrome is named after the blue color of a newborn’s skin when their blood nitrite levels are high. As a result, methemoglobinemia is often known as “blue baby syndrome,” and it is a life-threatening disease. When nitrite enters the bloodstream, it causes the hemoglobin (the protein that transports oxygen in the bloodstream to the body’s tissues) to be oxidized to methemoglobin. The Fe^2+^ of the hemoglobin group is oxidized to Fe^3+^ as shown in the following reaction [97,98]:

4Hb (Fe^2+^) O_2_ + 4NO_2_^−^ + 4H^+^ → 4Hb (Fe^3+^) + 4NO_3_^−^ + O_2_ + 2H_2_

This reaction produces methemoglobin which is responsible for the reduced oxygen supply to body tissues, causing the skin to become blue and possibly causing asphyxia. In the initial stages of methemoglobinemia, the blue color can be observed in the nose, lips, and ears and in extreme cases it can affect the peripheral tissues [45]. Infants under six months of age are the most sensitive to methemoglobinemia. Meanwhile, this disease has been reported in both school-going children and adults [99].

Furthermore, decreased tissue oxygenation can have a variety of negative consequences for the children, involving coma and eventually death. The toxic amounts of nitrites responsible for methemoglobinemia range from 0.4 mg to more than 200 mg per kg of body weight. The nitrite ion limit for newborns is up to 3 ppm [45]. The U.S. Environmental Protection Agency reported contradictory evidence over the relationship between higher nitrite intake and the elevated incidence of cancer in children and adults. In certain studies, it has been found that a high intake of nitrite can lead to the elevated occurrence of leukemia, nasopharyngeal and brain tumors in some children [45].

## 6. Potential Alternatives to Nitrite in Processed Meat and Their Effect on Color, Flavor, Antimicrobial and Antioxidant Properties

**Figure 4:**
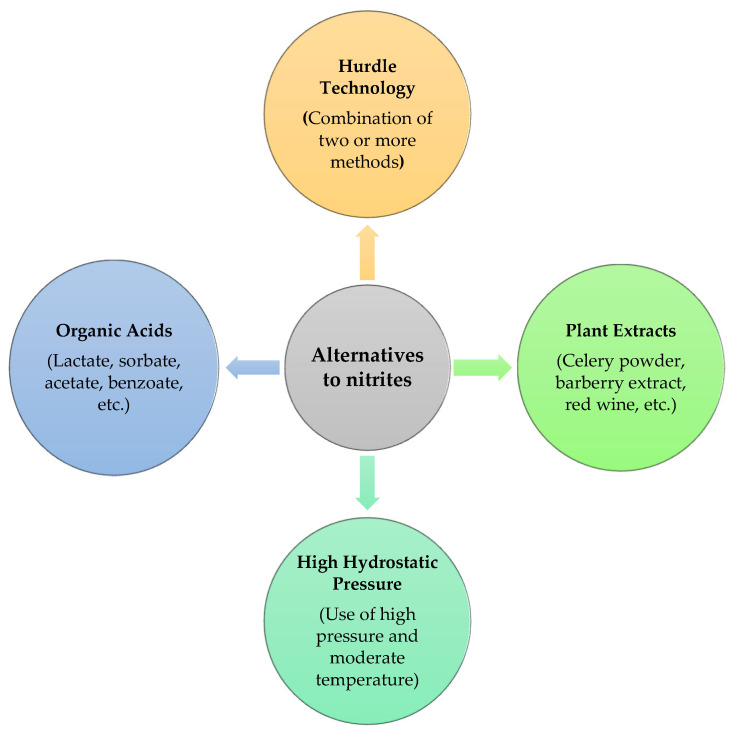
Alternatives to nitrite in processed meat.

As nitrite is involved in the production of nitrosamines, meat industries are recently focusing on new strategies to substitute traditional NaNO_2_ in cured meat with the aim of minimizing nitrite intake. Consumer’s interest is also growing in the development of natural alternatives and other preservation methods that are comparatively healthier. Although nitrite’s broad-spectrum action makes it hard to replace it with a sole antimicrobial agent, a mixture of nitrite and other antimicrobial agents might become effective [26]. Nevertheless, any improvements in terms of consumption safety should be made without compromising the distinctive features of the organic and natural processed meats, and this must be linked to the consumer’s desire to purchase such foods. It is possible that a replacement for nitrite might be found and new products may be developed, but it is questionable if this might be good enough to entice people to buy. Consumers prefer meat products which contain lower nitrite levels and the decision of buying new meat products depend on the function of nitrite, their application reasons and their outcome [100]. Therefore, a successful nitrite reduction in meat products, along with the addition of several alternatives (Figure 4), would provide a variety of benefits for the consumers, including a reduction in cancerogenic substances.

### 6.1. Plant Extracts

Nitrate is abundant in leafy green vegetables. Vegetables such as celery, lettuce, cress, spinach, rucola, etc., have been found to contain more than 2500 mg nitrate/kg [101]. As nitrate can be reduced to nitrite by several microorganisms, these vegetables can be utilized as a partial or whole alternative to chemical nitrite in meat curing.

Parsley (*Petroselinum crispum*) has a high nitrate content (1000–2500 mg/kg on average) and it can be used as an alternative to nitrite in processed meat [102]. Mortadella-type sausage produced with nitrite (greater than 60 ppm) from parsley extract powder appears to be equivalent to traditionally cured sausages in terms of *L. monocytogenes* inhibition and microbiological deterioration during storage. The microbial cell count of *L. monocytogenes* reduced as the percentage of added extract increased. By using parsley extract powder, reduced residual nitrite levels can be attained, and by marketing these meat products, customers’ nitrite intake can be reduced. Application of 4.29 g PEP/kg sausage meat (120 ppm nitrate) appeared to be equivalent to the conventional use of nitrite salt in mortadella-type sausages in terms of the red color during most of the storage period. Consumer acceptability for products made with greater quantities of parsley extract was found in a recent study, suggesting that these items might be commercial. Parsley is also less allergic than celery extract [102].

Celery can be a viable alternative to NaNO_2_ in cured or processed meats. The application of 0.8% celery powder for sausage production can meet the conventional standards. Sausages made with celery powder showed similar outcomes to the control (sodium nitrite) in terms of TBA, pH, total microbial count, VBN content, and sensory assessments. Furthermore, CP (celery powder) provided a more attractive meat color and reduced the residual nitrite content similar to the control as it naturally contains both pigment (betalains) and nitrate. The use of celery (as a concentrate or as a concentrate fortified with citric acid) seemed to have no effect on the product’s redness; the a* (redness) value was reported identical to the value of the samples made with NaNO_2_ [103]. The sensory qualities of sausages were unaffected by the addition of celery powder. As a result, celery powder improved the physicochemical characteristics of sausages during refrigerated conditions. A 75.6 g celery concentrate suppressed the multiplication of *L. monocytogenes* in a comparable way as NaNO_2_ at 100 mg/kg. However, the most effective outcome of the application of celery powder was observed when combined with 10% citric acid [103].

Spray-dried Swiss chard powder has recently been utilized as a natural nitrate source. This item is comparable to celery powder and it contains approximately 3.0–3.5% nitrate [104]. The color consistency of cooked pork patty was enhanced by adding Swiss chard powder (PS). The shelf life of the product was also extended due to the inhibitory actions of PS on coliform bacteria. PS also enhanced the redness value (a*) of the samples due to greater nitrosoheme pigment concentration. The production of nitrosoheme pigments was favorably influenced by the addition of pre-converted nitrite (2%) from Swiss chard powder. The acidic pH of PS also showed a reduced residual nitrite concentration in the cooked pork patties [105]. Moreover, PS prevented lipid oxidation in the cooked pork patties. This is possibly due to the presence of phenolic acids (antioxidant) and flavonoids, such as kaempferol and syringic acid [106]. As a consequence, cooked pork patties containing PS gained improved flavor and high acceptability ratings. The major benefit of Swiss chard powder is that it is free from allergens [104].

Barberry extract can be used as an alternative to nitrite in processed meat. In cooked sausages, the application of a high concentration of barberry extract (90 ppm) combined with a low concentration of nitrite (30 or 60 ppm) showed an increased shelf life under cold storage (4 °C). The antioxidant properties of the sample were improved by barberry extract addition, indicating that partial substitution of sodium nitrite with this plant extract might produce a healthier meat product [107]. Due to the antioxidant properties of barberry extract, it can be used in meat products instead of nitrite to prevent carcinogenic nitrosamine formation. In the sensory analysis, samples prepared with 90 ppm barberry extract and 30 or 60 ppm nitrite (lowest) gained the highest rank [107].

Red wine or red wine mixed with garlic, has been demonstrated to be a viable option for controlling biological hazard in the production of chouriço (dry-cured sausage). In a recent study, it was revealed that the addition of wine or wine and garlic increased *Salmonella* destruction during meat processing [2]. The color of chouriço prepared with wine or wine mixed with garlic was better preferred by the consumers. On the contrary, the samples produced with traditional nitrite salt was rated as artificial and over-cured. The use of 7.5% red wine to chouriço showed a high redness value (a*) and the same value was observed with the addition of 150 ppm sodium nitrite. Furthermore, 7.5% red wine mixed with 1% garlic enhanced the yellowness (b*) of the sausage due to the reaction between these two items [2]. The combined effect of these two ingredients also showed a slight brownish color in the product. Moreover, a strong cured flavor was exhibited in samples produced with red wine or a mixture of wine and other ingredients. Furthermore, chouriço prepared with red wine was better rated than samples made with only nitrite salt [2].

In a recent study, beetroot powder (*Beta vulgaris*) was used as a nitrite alternative in Turkish fermented sausage [108]. The beetroot powder exhibited a change in the quality traits of this new product. Depending on the quantity of the powder used, the redness value (a*) of the sausage changed. The researchers also reported that beetroot powder reduced the yellowness value and increased the lightness value during the early period of storage. The highest redness value was obtained from the sample formulated with 0.35% of the beetroot extract. The redness value was well conserved during the storage period when a higher quantity of beetroot extract was added. However, samples containing an increased amount of beetroot powder and a low amount nitrite/no nitrite exhibited a higher oxidation. Although having polyphenolic compounds, *B. vulgaris* did not reduce the TBARS values during the storage period. However, sensory analysis of the sausage formulated with different amounts of beetroot extract (0.35%, 0.24%, 0.12%) reported no change when compared to the control (150 ppm NaNO_2_) [108].

The effect of grape seed extract with olive pomace hydroxytyrosol, and chestnut extract with olive pomace hydroxytyrosol, on the color of cinta senese dry-fermented sausage was observed by some researchers. The addition of grape seed extract resulted in high redness values which was probably due to the effect of Zn-protoporphyrin formation [109]. The microbial counts of *L. monocytogenes*, *E. coli*, *Clostridium* spp., *Staphylococcus* spp. and *Salmonella* sp. were found in low numbers for up to 3 weeks of ripening. The researchers also found that these extracts can inhibit lipid oxidation as they contain polyphenols [109].

Tomato pulp powder (TPP) can be applied successfully to pork luncheon rolls at a concentration of 1.5% without affecting the physicochemical characteristics. The impact of tomato pulp powder on luncheon roll products was examined by some researchers [110], who discovered that the redness (a*) values and the yellowness (b*) values increased while the lightness (L*) values dropped. However, the preparation of luncheon rolls supplemented with 3% TPP (tomato pulp powder) would be unfeasible due to the negative physicochemical and sensory effects. The decrease in nitrite concentration from 100 to 50 mg/kg showed no adverse effects on texture or microbiological stability (TVC) and boosted the overall product acceptance. According to ANOVA and chemometric techniques, the pork luncheon roll made with 1.5% TPP and 50 mg sodium nitrite exhibited the same (*p* > 0.05) or improved sensory characteristics in comparison to the luncheon roll with no TPP and 100 mg/kg nitrite. In another study, cooked sliced pork luncheon roll formulated with 1.5% TPP mixed with lower nitrite showed better technological and sensory qualities. The interaction between nitrite and plant extract may lead to a pro-oxidant state. In luncheon rolls, a mixture of tomato pulp powder and nitrite has shown a pro-oxidant impact [110]. Moreover, tomato pulp powder gives the product a savory taste. Furthermore, tomato processing wastes, such as peels and seeds, contain various bio-active components such as carotenoids (b-carotene, lycopene, phytofluene, phytoene and lutein) [111]. Moreover, tomato contains important natural pigments which are used as coloring agents in various meat products [112]. In addition, tomato powder can retard the oxidation process and improve the consumer acceptability when used as a natural alternative to nitrite in frankfurters [113]. In addition, it has been reported to lower the residual nitrite concentration in frankfurters [113].

Grape pomace can be used as an alternative to nitrite in meat processing. Several physicochemical, microbiological, and sensory characteristics of beef sausages were affected by the addition of grape pomace. These findings show that adding large quantities of grape pomace (about 2%, *w*/*w*) as a partial replacement of nitrite might alter the color characteristics of sausages, resulting in a darker product. Nitrite-reduced sausage systems such as beef sausages prepared with 1 or 2% grape pomace and nitrite (30 mg/kg) showed an elevated yellowness of the products. On the other hand, grape pomace reduced the redness (a*) and lightness (L*) values of beef sausages in comparison to the control (120 mg/kg nitrite). On the first day of storage, sausages made with 2% grape pomace had higher oxidation values (*p* < 0.05). This was supported by a decrease in phenolic compounds and an elevation in total microbial counts after 30 days of storage at 4 °C [114]. Although there were changes in the sensory characteristics of beef sausages made with grape pomace, sensory panelists found these items to be acceptable. The addition of dry red grape pomace to sausages prevented bacterial growth during 1 month of cold storage. It also reduced residual nitrite levels, pH and extended the sausage’s shelf life. Furthermore, adding DRGP to the beef product increased the health-promoting aspects as well as stabilized the samples generated [114]. According to these findings, grape pomace powder can be used as a healthy food ingredient in diet formulation.

Pomegranate peel extract can be used as a natural curing agent in meat processing as it contains the highest quantity of polyphenols among other fruits (Table 1). Many researchers have investigated pomegranate peel as it has natural antioxidants including flavonoids and phenolic acids that have anti-cancer, anti-mutagenic, anti-cardiovascular disorder and anti-inflammatory properties [115,116]. Beef sausages prepared the highest quantity of PPE, pistachio green hulls (1250 ppm) and no added nitrite showed higher yellowness (b*) values than the control (where 120 ppm sodium nitrite was added) [117]. Another study reported that the use of PPE and pistachio green hulls decreased the MDA formation [118]. In compared to essential oils (EO) or synthetic antioxidants, PPE and PGHE are affordable, plentiful, and economical sources of phenolic content. They have a high TPC content and excellent antioxidant activity. The TBARS and PV values of samples containing less nitrite and more plant extracts were comparable or better than the control. Microbial analysis revealed that both extracts provided complimentary effects in keeping total viable count and harmful bacterial growth below the threshold level. Therefore, these plant extracts can be used with decreased levels of nitrite (up to 50%) in cooked sausages to improve functional characteristics and reduce the production of carcinogenic nitrosamines [118].

The seed of the annatto plant (*Bixa orellana* L.) has been used as a natural colorant in Asian cuisine. Among all natural colorants, annatto took second place in terms of economic value [119]. Annatto seed powder may be an effective alternative to nitrite in pork sausage production as it improves the color and textural properties of sausages. In a recent study, pork sausage made with various concentration of annatto seed powder (0.2%, 0.1%, 0.05%, 0.025%) reported an increased value of a* and b* parameters with an increasing amount of *Bixa orellana* (L.) seed powder. Furthermore, annatto added to sausages with 37.5 ppm nitrite was more effective in the lipid oxidation retardation process as well as in the inhibition the microorganism growth than the control sample (150 ppm nitrite). The greatest inhibition of TBARS formation was observed when the highest amount (0.2%) of the extract was used. The reduction of lipid oxidation products (primary and secondary) was observed to be comparable to that observed in the control sample (150 ppm nitrite) [120]. Therefore, annatto seed powder can be utilized for the substitution of nitrite in the preparation of meat products as a source of natural anti-microbial and antioxidant agents for extending the shelf life of the product.

### 6.2. Organic Acids and Salts

In meat industries, organic acids are used to prevent microbial development, decrease the p^H^ of meat products, and increase the curing performance of processed meats [36,122]. The use of organic acid to cured meat enhances the color development process while inhibiting microbiological growth. Lactate, sorbate, acetate, and benzoate are some important organic acids that have been widely used as food additives for many years. The rationale for employing organic acids is that they have the potential to lower pH to a level that prevents bacteria from proliferating [123].

Lactate (lactic acid salt) can be applied in the manufacturing of processed meats and it has been proven to improve color stability, flavor and shelf life. Although various organic acids might be employed as antimicrobial agent, lactate is the most effective among all since it has the ability to enhance the meat quality by imparting a salty flavor, while also maintaining color and contributing to a greater ability to hold water [124,125]. Adding a little quantity of sodium/potassium lactate, salts, and water to meat can enhance the flavor, color and tenderness while also delivering antibacterial and antioxidant properties [126,127]. When introduced to raw meat, sodium lactate prevents the formation of foul and rancid flavors and appears to be an effective flavor enhancer without any negative effects [128]. One possible mechanism behind the inhibition of off-flavors by sodium lactate treatment is the radical scavenging activity. It binds to the free radicals present in meat and prevents lipid oxidation. However, the mixture of sodium lactate and NaCl significantly decreases the oxidative reactions caused by NaCl, observed by a decrease in the TBA value (0.463 to 0.384). Ground pork treated with a combination of NaCl and sodium lactate exhibited a significant (*p* < 0.05) reduction in lipid oxidation when stored at 2 °C [129].

Lactate promotes the color stability of fresh meat through increased LDH (lactate dehydrogenase) activity. LDH converts lactate to pyruvate while reduced NADH is also replenished. This reproduced NADH then reduces metmyoglobin (Fe^3+^) to deoxymyoglobin (Fe^2+^). As deoxymyoglobin can reduce NO_2_ to NO [126], increased deoxymyoglobin generation is thought to result in increased nitric oxide production from nitrite and low residual nitrite remains in the samples. The NO produced from nitrite can then be combined with the residual deoxymyoglobin in the form of iron-nitrosylated myoglobin [130]. Through this mechanism fresh meat can maintain a pinkish-red color over an extended period of storage. In a previous study, researchers have observed that application of lactate in fresh meat products preserved the myoglobin pigments from being oxidized under oxidizing environments, allowing more red oxymyoglobin formation and retained the oxymyoglobin pigment for a longer period [131]. In another study, it was noticed that sodium lactate (1%) can retain the red cured color of the cured pork sausages while improving the salty flavor of the product. It was also shown that sodium lactate lessens taste loss and improves palatability during storage for every 1% increase in sodium lactate [132].

Lactate’s antimicrobial impact on meat has been researched, and its effectiveness against a variety of pathogenic microbes and diseases has been demonstrated. It is worth noting that as the addition of sodium lactate rises, the number of aerobic as well as anaerobic microbes reduces. The conversion of pyruvate to lactate may be altered by high amounts of lactate ions, which is similar to thermodynamic equilibrium [133]. Furthermore, sodium lactate salt works as a fragmented acid that penetrates through a bacteria’s membranes, creating an acidic intracellular state [134]. Experiments have shown that bacterial intracellular pH as well as cellular metabolism is rapidly reduced due to cell body denaturation and eventual cell death of the bacteria [135]. For this reason, sodium lactate addition is said to cause reduced water activity and thus prevent microbial development. The inhibition of *C. perfringens* growth by added Na or K lactate depends on the quantity added, type of product, moisture content, p^H^, adjunct salt and spice levels, as well as storage temperature [136,137,138,139]. Sodium lactate added to the sausages was demonstrated to improve the microbiological quality, provide a longer shelf life and enhance antimicrobial action compared to sausages treated with sodium nitrite [140]. The effect of sodium lactate and NaCl together (20 and 20 g/kg) or NaCl alone on the chemical and microbial quality of uncooked ground beef (at 2 °C storage conditions) were studied; sodium lactate in combination with NaCl or NaCl alone slowed the growth of APC (aerobic plate count), lactic acid bacteria, psychotropic count, Enterobacteriaceae, as well as prolonged the shelf life of the product up to 21 days and 15 days, respectively [141]. Sodium and potassium lactate also inhibited *Clostridium perfringens* development in processed beef goulash while stored under isothermal conditions [136]. The use of 2% sodium lactate in poultry sausages prevented aerobic psychotropic and lactic acid bacteria from growing during cold storage [142]. *Lactobacillus alimentarius* and other LAB (lactic acid bacteria) did not grow in cooked or acidified chicken meat for 14 days after being exposed to 3.3% sodium lactate. The total viable counts of frankfurters (low-fat) made with 2% sodium lactate was lower in comparison to the control (NaCl) after 28 days of storage under refrigerated conditions [140].

The most important impact of sodium lactate in meats is its capability to extend the shelf life. It has been observed that sodium lactate extends the shelf life of meat products such as ham and cured sausages by around 1 to 2 weeks. After two months of refrigerated storage, sausages treated with 1.8% sodium lactate revealed no signs of deterioration, but the control group, which did not employ a preservative, showed some signs of deterioration after 1 month [140]. The addition of 1% or 2% sodium lactate to sliced poultry sausages prolonged the shelf life by 3 or 4 times, respectively when stored at 5–7 °C [142]. The use of sodium-lactate salt in fresh pork-sausages was shown to be quite effective in reducing bacterial count and increasing the shelf life for around 14 days longer than the control experiment.

The application of potassium lactate in beef slices can enhance or retain the similar characteristic color, flavor, sensory taste, lipid oxidation, shear and cooking characteristics to conventionally processed beef patties [143]. The addition of 3% potassium lactate on the outer surface of frankfurters exhibited a slightly reduced water activity (a_w_); the sensory panelists, on the other hand, only saw a minor effect or no change in sensory characteristics [144].

Buffered sodium citrate or the mixture of buffered sodium citrate and sodium diacetate may be successfully utilized to prevent the proliferation of *C. perfringens* during the chilling of injected pork and roast beef. The addition of sodium citrate during roast beef preparation led to a reduction in the growth of *C. perfringens* by 2.47, 1.87, 0.98 log CFU/g at 2.0%, 1.0% and 0.5% concentrations, respectively [145]. Moreover, a similar experiment was conducted on ground turkey where two extra organic acids were used (sodium lactate and sodium acetate); 1% concentration of sodium lactate and sodium acetate impeded *C. Perfringens* germination as well as proliferation (less than 1.0 log CFU/g) [146].

Sorbate, propionate, and benzoate are commonly recorded as “safe additives” and these additives have been conventionally used to prevent the growth of mold in numerous foods [147]. These novel antimicrobial additives suppress the development of various Gram-positive bacteria such as *Staphylococcus aureus*, *Listeria monocytogenes* and *Clostridium botulinum* in meat systems [148,149]. Various research data in this regard has shown that 0.1% benzoate–sorbate and benzoate–propionate inhibited *L. monocytogenes* growth in some ready-to-eat meat products [150]. It is difficult to completely replace sodium nitrite with sodium sorbate as it cannot perform all of nitrite’s functions, such as color development. A possible alternative to sodium nitrite (120 ppm) alone is the combination of sodium–sorbate (2600 ppm) and sodium nitrite (80 ppm). When compared to nitrite–sorbate mixtures or nitrite alone, sorbate increased product rancidity. A high concentration (2600 mg/kg) of sorbate seems to affect (although not considerably) the sensorial quality of mortadella when compared to relatively low concentrations (1000 mg/kg) and nitrite alone [59]. The development of *Listeria monocytogenes* on the exterior of frankfurters was suppressed by a benzoate mixture at 25% (*w*/*v*) [151]. Furthermore, in broth tests with no salt, sodium benzoate (2000 ppm) showed an inhibiting effect on the growth of non-proteolytic *Clostridium botulinum* [152].

### 6.3. High Hydrostatic Pressure

Treatment with high pressure (100–800 MPa) is used uniformly to meat products at moderate temperature (less than 45 °C) as an anti-microbial process with the purpose of extending the shelf life of that product. HHP increases the meat product’s shelf life by reducing the growth of pathogenic microorganisms [153,154]. The use of HHP also aids in the inactivation of enzymes for a greater duration of time without the use of synthetic additives. However, in order to ensure food safety and to increase the shelf life, proper application of pressure and temperature has to be set in accordance with the product’s characteristics [155]. Meat processors can now satisfy the growing demand of consumers for natural and “preservative-free” meat products while retaining the stable sensory qualities over a longer storage period and ensuring product safety by processing meats using HHP [154,156,157].

The pressure used in HHP treatment is isostatic, which means that the pressure is distributed uniformly and instantaneously to the product. Moreover, it is an adiabatic process which implies that there is a little temperature difference with the rising pressure (the temperature rises by nearly 3 °C for every 100 MPa rise, based on the food properties) irrespective of the product’s size or shape [158]. The product which is to be treated is loaded into the pressure vessel, and the vessel is filled and sealed after covering the product with a fluid. This fluid, often water or a glycol solution, is used to transmit the pressure throughout the system. A pressure pump with extra fluid injection is used to support the system. The treatment efficacy is largely determined by the amount of pressure used and the time frame held [159]. The pressure level ranges from 100 to 800 MPa and the retention time varies from a few seconds to 20 min. As per the isostatic theory, consistent pressure is applied to food uniformly in every direction. After applying and releasing pressure, the product will revert to its original form. The process of microorganism inhibition by HHP is affected by microorganism species, time, pressure level, temperature, water activity, and food composition [160]. HHP seeks to preserve food in a mild manner while eliminating harmful and pathogenic microorganisms. The cell membrane of microorganisms is most vulnerable to pressure damage. Cells are damaged and lose their integrity because they are unable to regulate the passage of fluids and electrolytes through their membranes under pressure. As a result, they eventually lose their reproductive ability [161]. Generally, Gram-negative bacteria are more pressure resistant than Gram-positive bacteria [160].

The application of HHP treatments in salted white chicken meat develop the cooked meat texture and the color of ground beef. This data may be utilized to create HHP treatment as a processing option to minimize NaCl and nitrite application while retaining the physical properties, meat quality and functionality [162]. HHP is a strong strategy for minimizing *Salmonella* spp., *Campylobacter* spp., *Listeria monocytogenes* in sliced cured ham [74]. *Salmonella* spp. and *Campylobacter* spp. was not found in sliced cured ham when conducted with HHP at 600 MPa (31 °C) for 6 min, but *L. monocytogenes* was found in untreated samples (25 g) at the initial time. However, *Listeria monocytogenes* was not found in HHP-treated samples studied for 4 months at 4 °C [160]. A reduction (10 logs) in the most resistant strain of *Listeria monocytogenes* was found in pork sausages when treated with HHP at 400 MPa and 50 °C for 6 min [163].

HHP at 400 MPa, 17 °C for 10 min substantially decreased the levels of *Enterococcus* and *Enterobacteriaceae* in cooked sausages [164]. HHP has also been shown to retard lipid oxidation in pork meat exposed to 800 MPa and therefore extend the shelf life [165,166]. HHP treatment as a non-thermal processing approach can be an effective strategy for reducing *L. monocytogenes* in sliced vacuum-packaged chorizo. The use of HHP at 400 MPa for 2.5–16 min enhanced the sensory characteristics of Portuguese chorizo as well as the overall brightness, firmness, and cohesiveness [167]. In accordance with this investigation, HHP at 500 MPa for 5 min and 600 MPa for 3 min had no negative impact on the sensory characteristics of salchichón and salami, respectively [166,168].

HHP has a substantial impact on the microbiological qualities of vacuum-packed wieners under refrigerated storage. HPP at 600 MPa, 8 °C for 3 min had no detrimental influence on the processing parameters and helped to increase the shelf life of all wieners of up to 84 days in storage. At 56 days of storage, HPP-treated wieners were evaluated and were substantially higher in overall acceptability than non-HPP-treated wieners. Regarding flavor acceptability, the magnitude of variation between the mean rated like values of HPP- and non-HPP-treated was the greatest at 6.1 vs. 4.7, respectively [169]. As a result, HPP can significantly improve the sensory quality of products, such as sodium-reduced formulations containing organic forms of nitrite. Moreover, an APC and LAB count in HHP-treated wiener was found below the detection limit of 1.0 log CFU/g for all preparations during the 12-weeks of storage period. Much prior research on RTE products found similar reductions in microbial population after HHP, indicating that HHP can significantly increase the shelf life of vacuum-packaged meat products [169,170,171,172]. According to LAB populations, the use of HHP technique to vacuum-packaged sliced ham enhanced the product’s shelf life. In this investigation, HHP at 400 MPa for 15 min extended the product’s shelf life from 19 days to over 85 days. This result demonstrates unequivocally that HHP is a highly useful method for extending the shelf life of vacuum-packaged sliced ham [173]. Table 2 summarizes the uses of different high hydrostatic pressure treatments and their effect on various meat products.

In sliced cured meats, HHP treatment is a highly promising method of preservation [166]. HHP at 400 MPa, 17 °C for 10 min after fermentation also enhanced the microbiological quality of the fermented sausages, without compromising the product’s quality [178,179]. HHP treatment may be recommended as a finishing step in the preparation of low-acid fermented sausages with suitable starter cultures [164]. HHP reduces the *L. monocytogenes* population in RTE processed beef products. The viable *L. monocytogenes* populations remained below the detectable limit of the sample after a 600 MPa HHP treatment for 4 min [180]. These findings are similar to another study that found a (3.85 to 4.35) log decrease in *Listeria monocytogenes* counts on RTE beef products after HHP (600 MPa, 17 °C, 3 min) treatment [172]. Similarly, HHP at 600 MPa, for 3 min reduced the *Listeria monocytogenes* counts in RTE sliced ham by 3.9 to 4.3 logs [66]. Some researchers looked at the possibility of utilizing HHP to extend the shelf life and enhance the food-safety of refrigerated ready-to-eat meat products when prepared with sodium nitrite [181]. They discovered that treating products with HHP at 600 MPa, 20 °C for 3 min was enough to keep the numbers of aerobic, anaerobic bacteria, and lactobacilli below the detection level for 95 days when stored at 4 °C. Combination of nitrite (75 ppm) and 450 MPa HHP produced satisfactory frankfurters in terms of functional characteristics (color, texture, and water holding capacity) [182]. As a result, *L. monocytogenes* inhibition programs can include a combination of HHP and antimicrobial chemicals, such as nitrite.

Combining high pressure technology with other hurdle methods can create a synergistic effect from the two hurdles and can improve the inhibitory impact. The combined impact of HHP (200 MPa for around 10 min) and enterocin LM-2 (2560 AU/g) on the shelf life of sliced cooked ham during refrigerated storage has been studied. Application of HHP (400 MPa) and enterocin (256 or 2560 AU/g) showed a suppression in the development of *Salmonella enteritidis* and *Listeria monocytogenes* below the detection level in sliced cooked ham. Furthermore, an increased shelf life of sliced cured ham of up to 70 to 90 days was observed under refrigerated storage [177].

## 7. Conclusions

Nitrite is used as a versatile additive in the meat industry. It is liable for the pinkish-red color and unique flavor of cured meat products. It also acts as an antioxidant that prevents the development of a warmed-over flavor as well as a bacteriostatic effect that prevents the formation of botulinum toxins from *Clostridium botulinum*. Despite its many advantages in meat curing, sodium nitrite has been the subject of debate due to its probable carcinogenic impact on humans, according to various research. Ingesting too much nitrite can induce methemoglobinemia in children and raise the risk of developing colorectal cancer in adults. On the other hand, consumers’ desire for organic or nitrite-reduced meat keeps growing. As a result, the meat industry is now focusing on finding efficient ways for minimizing residual nitrite content in meat products and safer nitrite alternatives for the preparation of organic meat products. As nitrite replacements, various plant extracts, organic acids (lactate, sorbate, etc.) and HHP can be employed efficiently in processed meats. Unfortunately, still no sole alternative for nitrite has been found that can fulfil all of nitrite’s functions simultaneously. Hurdle technology using reduced levels of nitrite combined with other additives or processing techniques might have potential in producing the antimicrobial effects against the most prevalent microbial pathogens while also improving sensory characteristics. However, additional research is required to find a single alternative to nitrite that can be used to perform the nitrite broad-spectrum activities in a cost-effective way.

## Figures and Tables

**Table 1:** Effect of different plant-based alternatives of nitrites on meat products.

Additives	Meat Products	Effects	Reference
Parsley extract powder (PEP)	Mortadella type sausages	*L. monocytogenes* reduction, reduced residual nitrite level	[102]
Celery juice concentrate or powder	Ham slices	Control lipid oxidation, color development	[103]
Spray-dried Swiss chard powder	Cured pork loins	TBARS reduction	[105]
Barberry extract	Cooked beef sausage	Color development, potential antioxidant properties, negative interaction observed between nitrite and extract	[107]
Red wine or red wine + garlic	Chouriços cold-dried, smoked sausages	Color development, strong cured flavor, inhibitory properties against *Salmonella*	[2]
Beet root powder	Turkish fermented sausage	TBARS reduction	[108]
Tomato pulp powder	Pork luncheon roll, frankfurters	Control lipid oxidation	[110,113]
Pomegranate peel extract	Beef sausage	TBARS reduction, hydroperoxides reduction	[117]
Cranberry powder	Fermented sausage	Control lipid oxidation reduced growth of *L. monocytogenes*.	[121]
Annatto seed powder	Cooked sausages	Color development, TBARS reduction, control of bacterial growth	[120]

**Table 2:** Uses of different HHP treatments and their effect on various meat products.

Treatment	Products	Effects	Reference
HHP at 600 Mpa	Sliced dry cured ham	*L. monocytogenes* inhibition after 120 days at 4 °C.	[160]
HHP at 400 Mpa	Vacuum-packed sliced cured ham	Extended the shelf life of products	[173]
HHP at 400 Mpa	Cooked sausages	Reduction of *Enterobacteriaceae* and *Enterococci*	[164]
HHP at 400 MPa + potassium lactate	Cooked ham (sliced)	*Listeria monocytogenes* and *Salmonella* spp. inhibition for 12 weeks	[174]
HHP at 450 MPa	Iberian ham	*Listeria monocytogenes* population reduction after 60 days	[175]
HHP at 800 MPa	Pork meat	Lipid oxidation retardation	[166]
HP at 600 MPa	Dry-cured ham, marinated beef loin and cooked ham	*Salmonella enterica*, *L. monocytogenes*, *S. aureus* below the detection levelduring 4 months of storage	[176]
HHP at 400 MPa + enterocin	Sliced ham	Suppress the development of *Salmonella Enteritidis* and *L. monocytogenes*	[177]

## Data Availability

The data presented in this study are available upon request from the corresponding author.
